# Cannabis Indica—Cannabis Americana1Read at the meeting of the Pennsylvania State Veterinary Medical Association, Philadelphia, March 3, 1897.

**Published:** 1897-04

**Authors:** Charles Williams

**Affiliations:** Philadelphia


### CANNABIS INDICA—CANNABIS AMERICANA.[1]

By Charles Williams, V.M.D.,
PHILADELPHIA.

The *United States Pharmacopœia* recognizes the dried tops of the cannabis sativa, or common hemp-plant, as it grows in India and in our own country. The fluid-extract of this plant is a dark-green, resinous liquid of a peculiar narcotic odor and taste. Gunjah is the dried plant as sold in the bazaars of Calcutta. Churrus is the resinous exudate with the epidermis, etc., scraped off the leaves. Hashish is an Arabian preparation of the drug. The resinous principle, representing the active ingredient of the drug, is known as cannabis. It also contains a trace of volatile oil.

*Physiological Action.* Indian hemp is a deliriant narcotic,

[1] Read at the meeting of the Pennsylvania State Veterinary Medical Association, Philadelphia, March 3, 1897.

anodyne, and antispasmodic. Bhang is said by Finlay Dun to be widely used in India to stay the flagging spirits, and larger doses to produce pleasing, drowsy narcosis. Similar effects are said by the same author to be produced in horses: the flagging appetite is improved, a capacity for exertion increased, and restlessness overcome. Sir Robert Christison stated that "for energy, certainty, and convenience Indian hemp is the next anodyne to opium, and often equals it." Mr. Richard Rutherford, of Edinborough (says Finlay Dun), has used gunjah for several years for colic in horses, and "finds that it acts promptly and without any of the headache, delirium, or blocking of the bowels, as opium is almost sure to do. Horses receiving full doses soon become drowsy, hang the head, finally relax the ears, close the eyes, nod the head, and sway from side to side. If the drug is continued they lie down, stretch out at full length, and certainly evince every symptom of dreaming, for they will move the lips, wink the eyes, and work the limbs as a dog will do when dreaming by the fire; but I never remember their making any vocal noise. The effects pass off in half an hour to an hour, when the animal rises and usually begins feeding without any other consequent symptoms."

*Cannabis in Colic.* It is on this point that I wish to speak of the drug as of the greatest use to the veterinarian. I consider this drug is the anodyne *par excellence* in all our animals, for, as Finlay Dun says, "it gives no deleterious after-results, and an animal with a simple case of abdominal pain may work again in a few hours when relieved." In treating a case of colic I find about two drachms of the fluid-extract poured on the tongue and repeated every fifteen to twenty minutes are about right; some use less and others use more. I believe there is no danger in larger doses, but I think equally good results are derived from oft-repeated small doses. At all events you will save expense and relieve the animal about as quickly. If the animal's circulation is good, so that absorption takes place readily, and the extract is made by a reliable chemist, the animal should be under the influence in about thirty to forty minutes, and either lie down or doze off standing, after using only one-half an ounce to one ounce of extract. There are occasionally cases where it will be necessary to administer four ounces. But I can assure you, gentlemen, when it takes four ounces or more of a good preparation of this drug things will begin to look serious, and the vigilant practitioner will perceive long before he has gotten to this stage that there are other things required besides a simple anodyne, for all we claim of this drug is to make the animal comfortable till nature can remove the offending element without interfering with the action of the bowels. Of course, I do not wish to be understood as recommending this drug as a panacea for all colics without any other treatment, as some do for their favorites (barium chloride, for instance); but when we get excessive flatus we need something to check fermentation, and the trocar to relieve pressure, and when we get a congestive colic we need the fleam, with mustard to the sides and cannabis by the mouth, and so on. But I will repeat, when a simple narcotic and anodyne are required, cannabis indica is the drug *par excellence*, and I think if any of you have never tried it you should do so, and it will give you much satisfaction.

